# 2826. Epidemiology of Extended Spectrum Beta-lactamase (ESBL)-producing Enterobacterales and Antimicrobial Resistance in Urinary Tract Infection (UTI) and Acute Pyelonephritis (AP)

**DOI:** 10.1093/ofid/ofad500.2437

**Published:** 2023-11-27

**Authors:** Maria Fernandez, Meghan B Gavaghan, Amer Al-Taie, Gregory Stone, Ramy El Mahdy Kotb

**Affiliations:** Pfizer, Chapel Hill, North Carolina; Pfizer, Chapel Hill, North Carolina; Pfizer, Chapel Hill, North Carolina; Pfizer, Inc., Groton, Connecticut; Pfizer, Chapel Hill, North Carolina

## Abstract

**Background:**

Enterobacterales remain the most common cause of UTIs and are associated with increased rates of resistance to commonly prescribed antibiotics. The objective of this study is to describe the prevalence and frequency rates of ESBL-producing Enterobacterales (ESBL-E) causing UTIs and AP, as well as current antimicrobial resistance rates associated to these pathogens.

**Figure 1.**
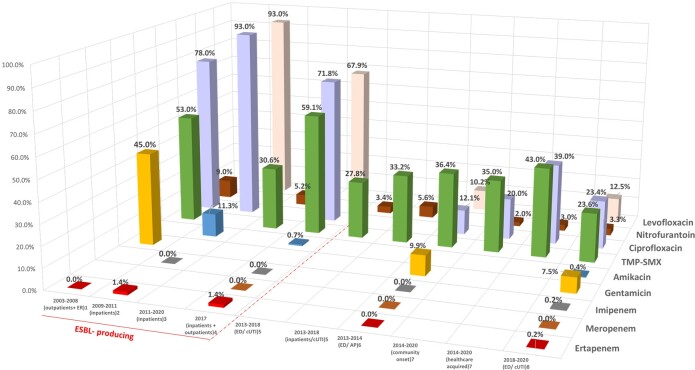
Percentage of E. Coli isolates resistant to antibiotics in the US. Red dashed line separates studies of ESBL-producing E. Coli isolates only from studies including all E. Coli isolates. AP = acute pyelonephritis; cUTI = complicated urinary tract infection; ED= emergency departments 1.Qi 2010; 2. Bouchillon 2013; 3. Aronin 2022; 4. Critchley 2019; 5. Lodise 2022; 6. Talan 2016; 7. Raphael 2021; 8. Faine 2022

**Figure 2.**
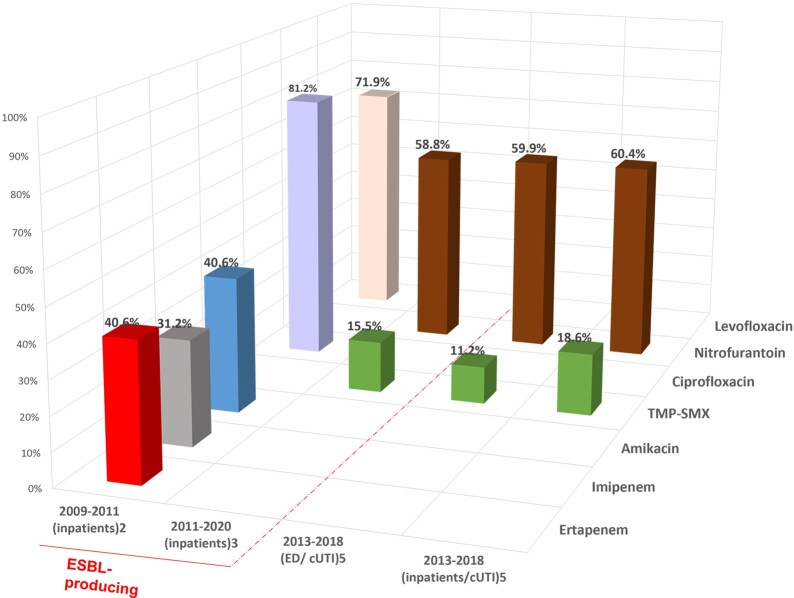
Percentage of Klebsiella pneumoniae isolates resistant to antibiotics in the US. Red dashed line separates studies of ESBL-producing Klebsiella pneumoniae isolates only from studies including all Klebsiella pneumoniae isolates. cUTI = complicated urinary tract infection 2. Bouchillon 2013; 3. Aronin 2022; 5. Lodise 2022

**Methods:**

A literature review in the PubMed database was conducted in July 2022. No date limits were included. Studies reporting ESBL-E prevalence or frequency rates and/or antimicrobial resistance rates from UTI and AP isolates in France, Italy, Germany, Spain, United Kingdom (UK) and the United States (US) were included. Studies of community acquired UTIs included outpatients and emergency departments; whereas those of healthcare associated UTIs included inpatients and long-term facilities.

**Results:**

Of 2,930 studies identified, 242 were selected for full-text review and 46 studies (10 from France, 7 from Italy, 11 from Spain, 2 from UK, 14 from the US, 1 from Europe and 1 from Europe + the US) met the inclusion criteria. During the last 20 years, ESBL-E rates increased in community acquired UTIs (from 1.1% to 4.2% in France; from 1.3% to 10.7% in Italy, from 2.8% to 13.9% in Spain, from 0.2% to 16.3% in the US) and healthcare associated UTIs (from 7.7% to 13.6% in France; from 1.5% to 21.5% in Italy, from 8.2% up to 12.2% in Spain, from 4.0% to 25% in the US). The highest ESBL-E rates were found in specific groups (25% in patients in nursing facilities, 27.4% in patients with AP/sepsis, 36.2% in patients ≥ 65 y). For some ESBL-E, high resistance rates to common antibiotics were reported: 56%-100% to ciprofloxacin and trimethoprim-sulphamethoxazole (TMP-SMX) in the European region, and in France, Italy, Spain, and the UK; 62% and 86% to gentamicin in the UK and Italy, respectively; 54% to fosfomycin in Spain; 54% and 76% to nitrofurantoin in Spain and the UK, respectively; 100% to levofloxacin in Italy and the European region. A resistance rate of 69.2% to imipenem was reported in Europe. Antimicrobial resistance rates in the US are depicted in Figures 1-2.

**Conclusion:**

Increased rates of ESBL-Es in community- and hospital-acquired UTIs, along with high resistance rates to many antibiotics, including carbapenems, were observed in Europe and the US.

**Disclosures:**

**Maria Fernandez, PhD, MBA**, Pfizer: I am an employee|Pfizer: Stocks/Bonds **Meghan B. Gavaghan, MPH**, Pfizer: Advisor/Consultant **Amer Al-Taie, MSc**, Pfizer Inc: Stocks/Bonds **Gregory Stone, PhD**, Pfizer: Stocks/Bonds **Ramy El Mahdy Kotb, MD**, Pfizer: Stocks/Bonds

